# Supplementary material for: Probing the causal role of prestimulus interregional synchrony for perceptual integration via tACS
Source: Sci Rep. 2016 Sep 12;6:32065. doi: 10.1038/srep32065 (PMC5018857; doi:10.1038/srep32065)
Supplement: Supplementary Information [file srep32065-s1.pdf]

Probing the causal role of prestimulus interregional synchrony for perceptual integration via tACS

Rolandas Stonkus[1,2], Verena Braun[2], Jess Kerlin[2], Gregor Volberg[3], Simon Hanslmayr[2]

[1]Vilnius University (Department of Neurobiology and Biophysics)

[2]University of Birmingham (School of Psychology)

[2]University of Birmingham (School of Psychology)

[3]University of Regensburg (Department of Experimental Psychology)

Address correspondence to rolandas.stonkus@gmail.com or s.hanslmayr@bham.ac.uk

Supplementary Material and Results

The median intensity ratings for the stimulation conditions are reported below. Since the data did not fulfil the criteria for parametric testing, non-parametric ANOVAs (Friedman ANOVAs) were carried out to test for significant differences in the ratings for pain, phosphenes and other visual artefacts. Concerning pain ratings, stimulation conditions triggered stronger pain than sham ($p<0.001$). Concerning phosphenes, no significant difference between conditions was observed ($p>0.15$). Visual artifacts were strongest in the Out-of-Phase condition, compared to the other conditions ($p<0.01$).

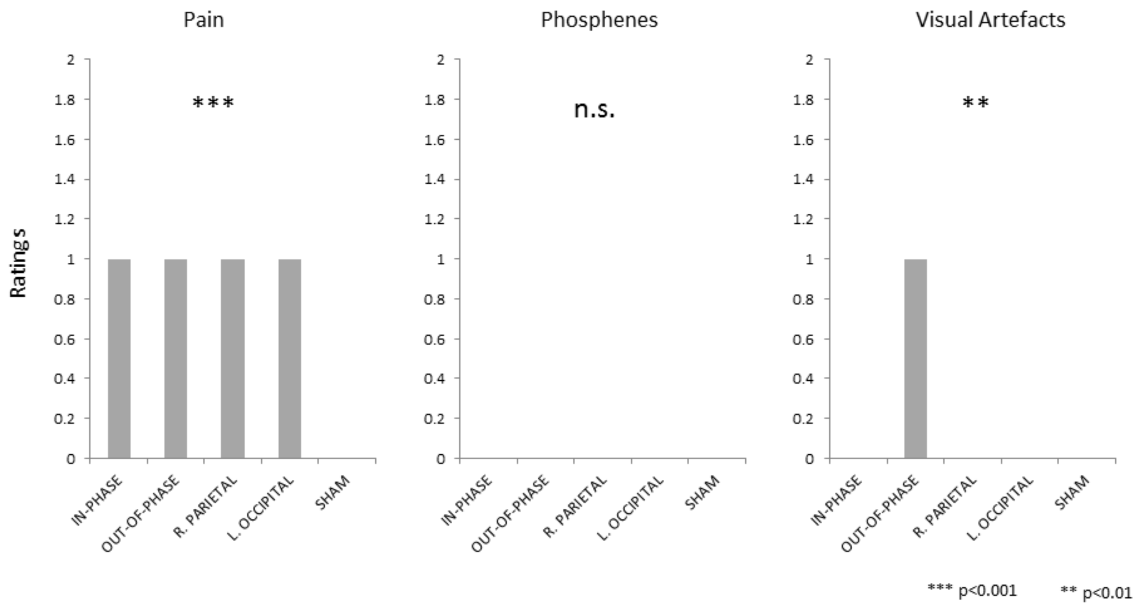

Supplementary figure 1. Median intensity/sensation ratings for pain, phosphenes and visual artifacts subjects are shown for the five different stimulation conditions.

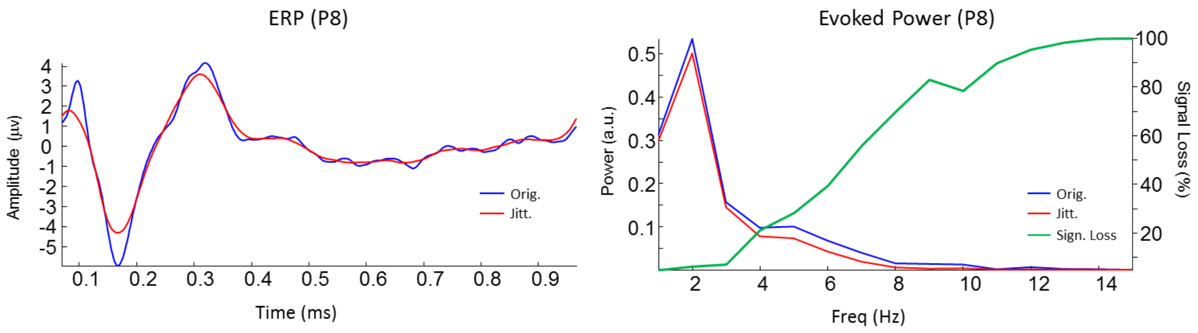

Supplementary figure 2. The results of a simulation are shown which demonstrate the effect of a jitter on visual ERPs. For the simulation the grand average ERP (blue line) from the sham condition was extracted and randomly jittered around +/- 35 ms (red line). This was done 100 times and an average ERP was calculated

across these randomly jittered ERPs. The results of the simulation for an exemplary electrode (i.e. P8, where entrainment echoes were visible) are shown. The left panel shows the visual ERP for the original (blue) and jittered (red) waveforms. It is evident that the jitter reduces the peaks and smoothes the waveforms (i.e. acts as a low pass filter). The right panel shows the ERP power of the jittered (red) and original (blue) ERPs, as well as the percent signal loss (green) due to the jitter. These results show that the jitter mostly reduces high frequency power, while less affecting the lower frequencies.
